# Specific analysis of highly absorbing nanoporous powder by small-angle X-ray scattering

**DOI:** 10.1107/S1600576722006987

**Published:** 2022-09-06

**Authors:** Zijie Lu, Diane Rébiscoul, Theyencheri Narayanan, Thomas Zemb

**Affiliations:** a Institut de Chimie Séparative de Marcoule, UMR CEA/CNRS/UM2/ENSCM 5257, BP17171, Bagnols-sur-Ceze, 30207, France; b ESRF, 71 Avenue des Martyrs, Grenoble, 38000, France; Australian Centre for Neutron Scattering, ANSTO, Australia

**Keywords:** small-angle X-ray scattering, highly absorbing porous powders, reflectivity and scattering contributions, absolute scattering intensity

## Abstract

The characterization of nanoporous powders of highly absorbing compounds by small-angle X-ray scattering (SAXS) involves overcoming several difficulties before quantitative information related to the porous texture, such as the specific surface and the porous volume, can be derived. In this article, first, the contribution of the grain facet reflectivity and scattering from the bulk of a grain with the density of ThO_2_, a highly absorbing material, were calculated. Microporous ThO_2_ powder having micrometric grain size was characterized, in which the scattering signal is predominant. A high-resolution synchrotron instrument was used in order to cover a wider *q* range and minimize the absorption effect, and the results were compared with those obtained using a laboratory X-ray source. Concerning the absorption problem existing with a laboratory X-ray source, a new and robust experimental method was proposed to correctly determine the scattering intensity of the highly absorbing granular samples on an absolute scale. This method allows one to calculate accurately the porous volume and the specific surface via Porod’s law and the invariant using a laboratory SAXS instrument. This last result opens new perspectives for the characterization of the volume and the specific surface of highly absorbing actinide oxide powders.

## Abstract 2

The contributions of grain facet reflectivity and scattering from the bulk of a grain of highly absorbing material were calculated as a function of sample characteristics. To test these results, the scattering intensity of a grain of microporous ThO_2_ was experimentally characterized by small-angle X-ray scattering using a synchrotron source. In addition, a new experimental method to probe the porous texture of nanoporous powders of highly absorbing compounds using a laboratory X-ray instrument has been proposed.

## 1. Introduction

The characterization of porous materials using small-angle X-ray scattering (SAXS) has a long history (Glatter & Kratky, 1982 ▸; Schmidt, 1995 ▸), but applications to nanoporous powders have developed in more recent decades (Spalla *et al.*, 2003 ▸; Chavez Panduro *et al.*, 2012 ▸). The principal advantage of this technique is that it probes the overall porosity, including the inner porosity often inaccessible with characterization methods using fluid intrusion. Indeed, fluid intrusion can be limited by the pore accessibility and the fluid molecules’ interaction with the surface, leading to an underestimation of the porosity (Okolo *et al.*, 2015 ▸).

From SAXS analysis, the pore volume and specific surface of the material can be obtained using different approaches, such as the invariant and Porod’s law (Spalla *et al.*, 2003 ▸; Chavez Panduro *et al.*, 2012 ▸; Né & Zemb, 2003 ▸; Cambedouzou & Diat, 2012 ▸). These methods can only be applied using the scattering intensity on an absolute scale. For granular media, the calculation of the absolute intensity requires a specific methodology clearly described by Spalla *et al.* (2003 ▸). However, this method cannot be applied directly to granular nanoporous media constituting highly absorbing compounds, which is particularly the case of nanoporous materials prepared with transition metal, rare earth, lanthanide and actinide elements. For the associated oxides at 17.4 keV, which is the energy of the Mo source classically used for SAXS instruments, the specific linear attenuation coefficients are between a hundred and a thousand cm^−1^ corresponding to absorption lengths of a few tens of micrometres. In this case, two experimental scenarios could be encountered: (1) the X-ray beam probes the compact zone of the granular (Glatter & Kratky, 1982 ▸) sample and (2) the X-ray beam probes the powder/air (non-compact) interface of the sample. In the first scenario, the scattering signals would be attenuated extensively, even for a sample with a thickness of 1 mm. In the second scenario, apart from the scattered photons, the outgoing beam collected by the detector might contain a part of the ‘leaked’ beam, *i.e.* the incoming photons that do not encounter any powder in the sample cell, and a part of the reflected beam due to the reflection of the powder at the powder/air interface. Regarding these possible experimental situations with highly absorbing materials, it is of particular importance to take into account all of these scattering processes to determine the real absolute scattering intensity. For this purpose, it is required firstly to distinguish the three components of the measured intensity, *i.e.* the leaked beam, the scattered beam and the reflected beam, and secondly to correct the absorption effect.

In this context, the goal of this article is to propose a new method that overcomes the difficulties described previously to obtain the scattering intensity on an absolute scale (Guinier & Fournet, 1955 ▸) and to quantify useful information related to the porous texture that has never been determined directly before for highly absorbing materials, such as the specific surface and the porous volume.

In the following section, the measured scattered intensities are theoretically described considering the different contributions coming into and out of the sample. The mathematical expressions of the scattered and reflectivity fractions of the measured intensity are established for one single spherical grain. Using these two mathematical expressions, several experimental cases with various highly absorbing grain properties, size and specific surface were calculated in order to highlight the predominance of the scattering or reflectivity signal on the simulated measured intensity. We have characterized microporous ThO_2_ powder with micrometric grain size as a model material, in which the scattering signal is predominant, using high-resolution synchrotron SAXS and a laboratory instrument. We propose a new and robust experimental method to correctly determine the scattering intensity on an absolute scale for highly absorbing granular samples. From the obtained absolute intensity, the specific surface and the porosity of the materials were determined using Porod’s law and the invariant method.

## 2. Theory

### 2.1. General formalism of small-angle scattering: case of a thin film

The scattering intensity is generally normalized in ‘absolute’ units to access quantitative information. The absolute intensityere 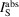 , expressed in cm^−1^, is given by

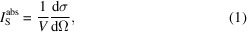

where *V* is the volume of the illuminated material and 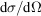 is the differential scattering cross section.

**Figure 1:**
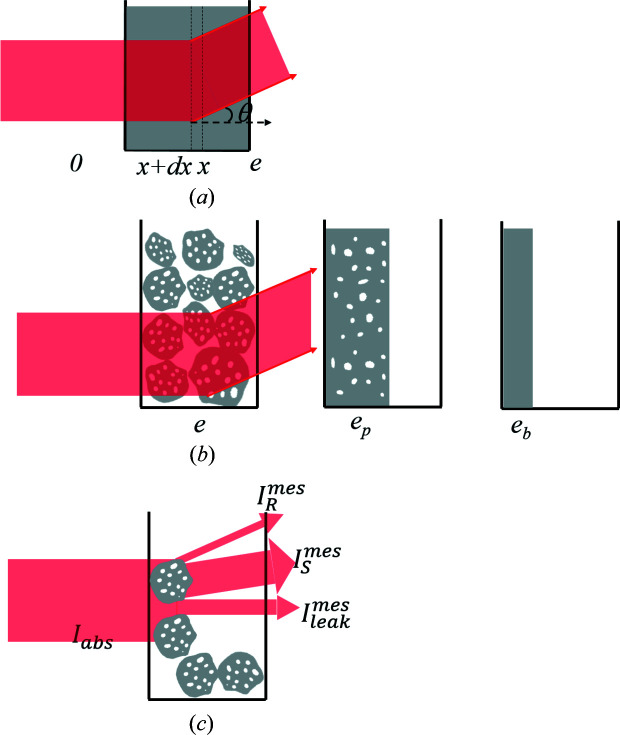
A representation of the X-ray beam pathway through three different types of samples. (*a*) A layer of dense material of thickness *e*; (*b*) a powder sample of thickness *e* with monodisperse porous grains, where *e*_p_ is the equivalent thickness of the porous material and *e*_b_ the equivalent thickness of the dense material obtained from the transmission coefficient *T*; and (*c*) a highly absorbing powder sample with monodisperse porous grains with significant voids in the bulk.

In general, SAXS experimental setups are designed for non-granular samples. For a sample with a thickness *e* [Fig. 1 ▸(*a*)], we assume that the incident flux is scattered at position *x* (0 ≤ *x* ≤ *e*) with an angle θ. Before scattering within the sample, the incident flux is attenuated by a factor exp(−μ*x*), with μ being the specific linear attenuation coefficient. After the scattering process, the scattered flux is attenuated within the sample by a factor 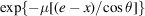 . Integrating the scattered beam over *x*, the experimental intensity at a scattering angle θ is obtained. The relation between the measured intensity 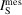 (the number of photons recorded by the detector) and the differential scattering cross section per unit volume of the sample *V* is defined as

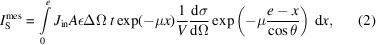

where *J*_in_ is the incident X-ray flux (counts per second per surface unit), *A* is the normal sample area exposed to the beam, ε is the detector efficiency, ΔΩ is the detection solid angle and *t* is the measuring time (s). For small-angle scattering, cosθ ≃ 1. Thus, 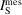 can be simply written as

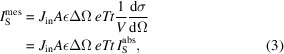

where *T* = exp(−μ*e*) is the transmission of the sample.

### 2.2. Case of granular porous materials

For a granular and porous system with inter-granular voids and intra-granular porosity as described in Fig. 1 ▸(*b*), the acquisition of quantitative information is more complex since the compactness and the porosity are unknown. In the work of Spalla *et al.* (2003 ▸), the normalization formalism was modified, transforming the granular materials into a continuous porous layer with a thickness *e*_p_ or a dense layer with a thickness *e*_b_ when the solid composition is known. Equation (3) can be then rewritten as

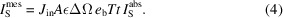

In practice, the sample transmission *T* can be determined by measuring the ratio between the direct beam through the sample and that without the sample. Knowing the linear attenuation coefficient μ_b_ of the solid part of the porous material, the apparent thickness *e*_b_ can be deduced by

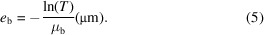




### 2.3. Case of highly absorbing granular porous materials

An important consideration when studying high-contrast materials is that the Rayleigh–Gans–Debye (RGD) approximation, (2π/λ)*d*|*n*−1| 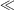 1, is not strictly valid, in particular in the low-*q* region probing the larger size scales. Here *d*, *n* and λ are the characteristic size, the refractive index and the wavelength, respectively. At the same time, a full Mie treatment is too complex for the present purpose (Van de Hulst, 1981 ▸). Since |*n*−1| 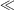 1 and *d* 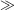 λ, the condition for the anomalous case involving a mixture of scattering, reflection and refraction (Van de Hulst, 1981 ▸) is satisfied. As a result, the measured intensity may comprise scattering, reflection and refraction contributions. The latter is manifested by the beam-broadening effect as presented in the supporting information. The scattering and reflectivity contributions are estimated in the following sections. But in the *q* and size ranges used for the calculation of nanoporosity (*q* > 0.1 nm^−1^), the phase shift [*p* = 2π*d*(*n* − 1)/λ] is still 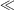 1 and the RGD approximation is valid.

When SAXS measurement is performed on highly absorbing powder with a grain size smaller than the beam size, typically lower than 1 mm^2^ [see Fig. 1 ▸(*c*)], equation (4) is no longer valid. The formalism developed by Spalla *et al.* (2003 ▸) takes into account an incident flux crossing a solid of an equivalent thickness *e*_b_ determined by the measured transmission. For highly absorbing materials, the measurement of an acceptable transmission is often complex and, thus, the determination of *e*_b_ is difficult. Indeed, as depicted in Fig. 1 ▸(*c*) with highly absorbing powder, the measured transmission of the sample depends on the thickness and the distribution of the grains. Two cases have to be considered: (i) when the beam is centered at the compact region, the thickness of the powders is high, which leads to a low transmission where the indirect absorption effect cannot be neglected; and (ii) when the beam is centered at the powder/air interface to measure a sufficient transmission, the intensity measured by the detector may include the leaked beam, which does not encounter any material. Moreover, the reflection of the beam at the surface of the grains also has to be considered. Consequently, the measured intensity *I* ^mes^ (counts) consists of three components:

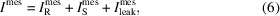

where 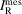 and 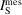 correspond to the reflected and scattered intensities, respectively, and 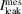 is the fraction of the incident beam that does not interact with the sample.

The measured transmission *T* ^mes^ can be expressed as

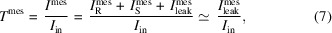

where *I*_in_ is the intensity of the incident beam.

To distinguish the scattered from the reflected signal, which is a difficulty also encountered with grazing-incidence small-angle X-ray scattering (GISAXS) (Lee *et al.*, 2005 ▸), the scattering has to be expressed in the same units as the reflectivity. In practice, scattering intensity is expressed as differential cross-section density, *i.e.* cm^−1^ (Né & Zemb, 2003 ▸), while reflectivity is expressed as the ratio between the reflected and the incident photon fluxes (Van der Lee, 2000 ▸; Daillant & Gibaud, 2008 ▸).

To the best of our knowledge, two practical methods are currently used. In the first method, which is used in the case of hard X-rays [e.g. ID10 at ESRF (Panduro *et al.*, 2014 ▸)], interference of reflected and scattered intensities is taken into account by calculating the intensity from the amplitudes [*A*(*q*) = *A*_reflected_ + (ρ_object_ − ρ_matrix_)*P*(*q*)*F*(*q*)*L*(*q*) with *A*_reflected_ being the amplitude of specular reflection, ρ_objec_ and ρ_matrix_ being the electron densities of the repeated objects and the matrix, respectively, *P*(*q*) being the form factor, *F*(*q*) being the structure factor and *L*(*q*) being the Laue function. The second method, independent of X-ray energy, is used in the case of GISAXS signal being always intimately mixed with specular reflectivity in any experiments (Naudon & Thiaudiere, 1997 ▸). Moreover, SAXS may also be present in practical cases. These three contributions are affected differently by the absorption effect occurring along different path lengths in the absorbing samples (Brumberger, 2013 ▸) and must be carefully evaluated in quantitative analysis. In the following section, expressions for the scattered and reflected intensities are proposed for one spherical grain and normalized to the same unit.

#### Determination of the scattered intensity 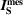


2.3.1.

**Figure 2:**
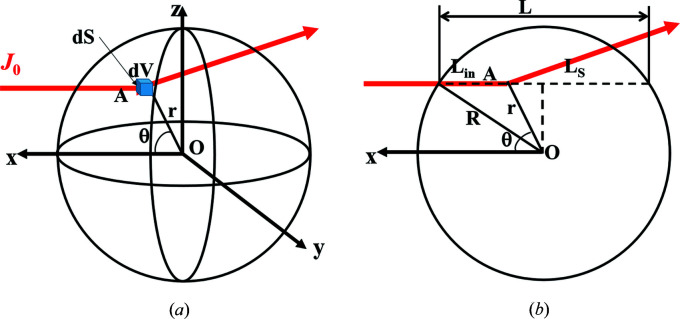
Scattering by unit volume d*V* at point A inside the sample, where OA = *r*, *R* is the radius of the grain, and *L*_in_ and *L*_S_ represent the attenuation length of the flux before and after the scattering, respectively.

For an infinitesimal unit volume d*V* [Fig. 2 ▸(*a*)], the relation between the observed scattered intensity 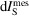 (counts) and the absolute intensity 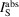 (cm^−1^) can be written as

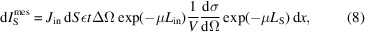

where d*S* is the unit surface perpendicular to the incident flux, and *L*_in_ and *L*_S_ represent the attenuation length of the flux before and after the scattering, respectively. For small angles, we can assume that *L*_in_ + *L*_S_ ≃ *L*, where *L* is the length of the chord indicated in Fig. 2 ▸. In this case, equation (8) can be rewritten as

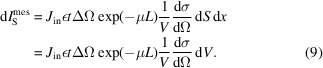

Integration over the sample leads therefore to the scattered intensity 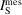 collected by the detector:

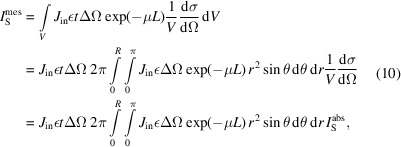

with *L* = 2(*R* ^2^ − *r* ^2^sin^2^θ)^1/2^, where *R* is the radius of the grain.

Equation (10) can also be rewritten in a similar form to equation (3):

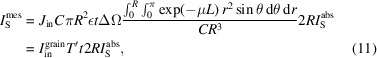

where 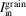 is the incident intensity illuminating the grain with normalization factor *C* and 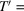 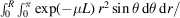 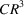 is the corrected transmission. By definition, *T*′(*R* → 0) = 1: 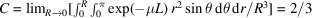 . Dividing 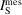 by the incident intensity 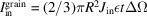 , we can now define the scattered intensity 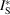 :

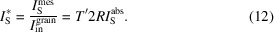




#### Determination of the reflected intensity 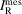


2.3.2.

**Figure 3:**
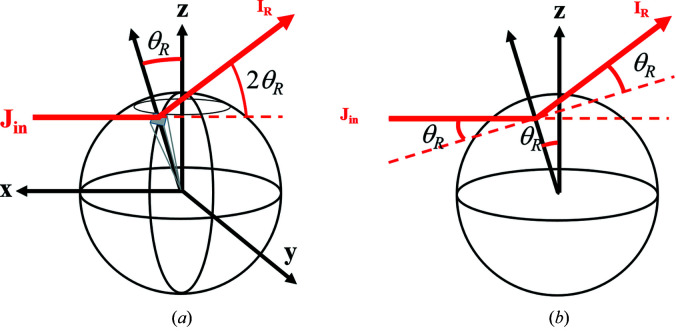
Reflectivity at a reflection angle θ_R_ on the unit surface d*A* of a grain with a radius *R*.

Fig. 3 ▸ presents the incident flux reflected on an infinitesimal surface d*A* of a grain with a radius *R*. Since the incident flux is considered a point source, no footprint correction is required. Thus, the reflected intensity on this surface 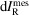 can be expressed as

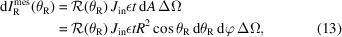

where 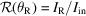 is the ratio between the reflected intensity and the incident intensity on the surface d*A* at the reflected angle θ_R_.

By integrating over the azimuthal angle φ, the total reflected intensity measured by the detector at the reflected angle θ_R_ is obtained:

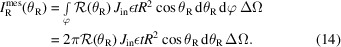

To remove the dimension, the reflected intensity 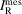 can be divided by 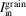 :

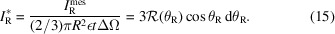

Since the reflectivity 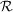 has three characteristic regimes (with the reflectivity wavevector transfer 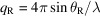 ), it is possible to write it as follows:

(*a*) When θ_R_ < θ_C_, 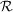 = 1.

(*b*) When θ_C_ < θ_R_ < 3θ_C_, 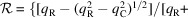 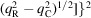 .

(*c*) When θ_R_ > 3θ_C_, 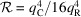 .

Here 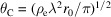 is the critical angle, with ρ_e_ being the electron density and *r*_0_ being the electron radius, and *q*_C_ is the wavevector transfer for θ_C_.

In this case, the reflected intensity can be expressed as

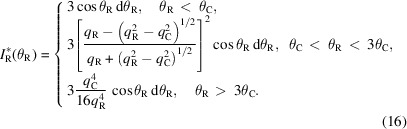

By considering cos θ_R_ ≃ 1 and *q*_R_ = *q*_S_ = *q* ( 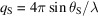 and θ_S_ = θ_R_ = θ), with λ being the X-ray wavelength and θ_S_ being the scattering angle, we obtained 

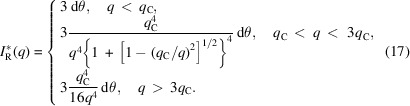




## 3. With which sample characteristics can the reflected signal be neglected?

The first question while performing an experiment is to determine if reflectivity 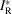 or scattering 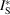 is dominant in the signal.

### 3.1. Calculation of the dimensionless intensity I* for one porous grain

To calculate the scattered intensity 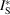 and the reflectivity 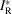 , three properties of the powder have been considered: the absorption length of the material (1/μ), the grain radius (*R*) and the internal Porod length (1/Σ) (Méring & Tchoubar, 1968 ▸), intimately related to the mean chord length (Gille, 2000 ▸; Méring & Tchoubar, 1968 ▸). In the following calculations, the influence of *R* and 1/Σ was determined on the contribution of 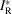 and 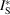 to the measured intensity 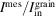 .

Here, 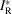 and 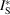 were calculated for one grain with a radius *R* between 1 and 200 µm, with Σ_pore_ (m^−1^) being the pore specific surface from 1.9 to 190 µm^−1^. By considering that the pores are cylindrical, we can deduce the density of the pores, *n*_pore_ (m^−3^), and calculate 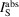 for the grain:

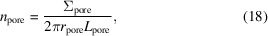

where 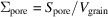 , with *S*_pore_ (m^2^) being the total pore surface area, *V*_grain_ being the volume of the grain (m^3^), *r*_pore_ (m) being the radius of the pore and *L*_pore_ (m) being the length of the pore.

In this case:

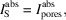

where

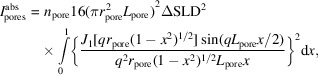




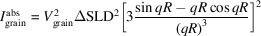

and

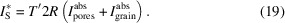

In these calculations, the solid part of the grain was considered as ThO_2_ as an example, and thus a linear attenuation coefficient of 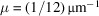 for 17.4 keV and a difference of scattering length density of ΔSLD = 6.82 × 10^11^ cm^−2^ were taken into account.

In addition, for these calculations, dθ was determined by the size of the detector pixel:

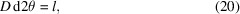

where *D* (m) is the distance between the grain and the detector (here *D* = 770 mm), and *l* (m) is the size of a pixel (here *l* = 150 µm). Thus, dθ was estimated to be 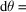 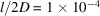 .

**Figure 4:**
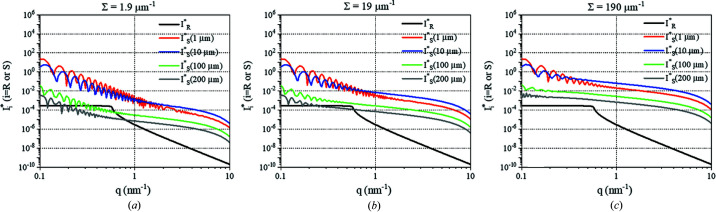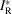 and 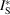 of one grain having various radii *R* and different pore specific surfaces Σ_pore_: (*a*) 1.9 µm^−1^, (*b*) 19 µm^−1^ and (*c*) 190 µm^−1^. The calculations were performed for a linear attenuation coefficient μ = 1/12 µm^−1^ and a difference of scattering length density of ΔSLD = 6.82 × 10^11^ cm^−2^.

The results are presented in Fig. 4 ▸. From these results, two ranges can be considered:

(i) 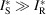 : when *R* ≤ 10 µm or Σ = 190 µm^−1^, the scattering is predominant over the reflectivity. In fact, the scattering intensity is related intimately to the specific surface of the grain. For a grain with a small radius, its external specific surface is big enough to have a high scattering intensity. For a grain with a large radius, the reflectivity signal can be neglected only when the grain is porous enough to provide more internal specific surfaces and increase the scattering signal.

(ii) 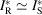 : when *R* ≥ 100 µm and Σ ≤ 19 µm^−1^, the reflectivity signal cannot be neglected compared with the scattering signal. The increase of the grain size leads not only to a smaller transmission but also to a smaller external specific surface. In this case, if the grain is not sufficiently porous to provide enough internal surfaces, the scattering signal is no longer predominant over the reflectivity signal.

These results highlight that, depending on the size of the grain and its internal specific surface, the reflectivity signal can contribute to the measured intensity. This adds complexity to the determination of the scattering intensity, which is required to quantify correctly the specific surface and the porous volume. This reflectivity contribution increases with increasing size and density of the grain.

### 3.2. Experimental scattering signal of one grain of microporous ThO_2_

**Figure 5:**
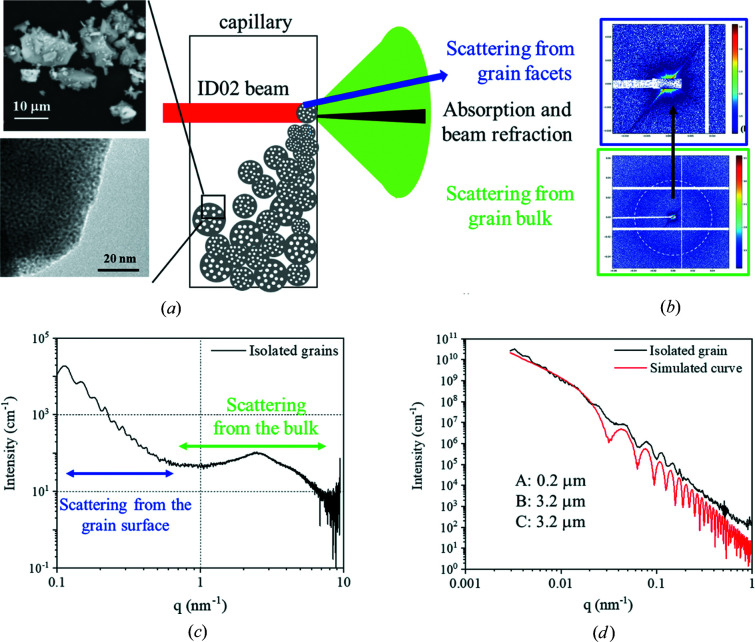
(*a*) A schematic illustration of the experiments, and images of the powder and of a part of a grain obtained by scanning and transmission electron microscopies, respectively; (*b*) a 2D SAXS pattern of the isolated grains; (*c*) an azimuthally averaged 1D SAXS pattern of the isolated grains; and (*d*) a selected sector-averaged (±1°) 1D SAXS profile of the isolated grain and the related simulated scattering curve of a rectangular cuboidal grain. The edge lengths are A = 0.2 µm, B = 3.2 µm and C = 3.2 µm.

To collect the experimental scattering signal, a capillary filled with ThO_2_ micrometric powder with organized microporosity was characterized using synchrotron SAXS on the ID02 beamline at ESRF (Grenoble, France) (Narayanan *et al.*, 2022 ▸). This powder with a grain size lower than 10 µm was chosen to avoid any reflectivity contribution in the collected signal. This material was synthesized by the colloidal sol–gel route and thermal treatment. During this thermal treatment, nanoparticles assembled to form a 3D bicontinuous porous network, as illustrated in Fig. 5 ▸(*a*). The operating beam energy was 16 keV with a beam size of 70 × 90 µm (vertical and horizontal, respectively). The sample-to-detector distances were 1.2, 6 and 31 m. The scattering patterns were recorded using an Eiger2 4M detector (Dectris). First, we centered the beam just above the powder/air interface of the capillary in order to probe just one or several isolated grains on the wall of the capillary. Figs. 5 ▸(*a*) and 5 ▸(*b*) present a schematic illustration of the experiments and the 2D SAXS pattern of the illuminated region, respectively. The scattering ring present in Fig. 5 ▸(*b*) is assigned to the scattering of organized micropores in a few grains, while the spike-like patterns near the beam center [Fig. 5 ▸(*b*) top] are due to the scattering from the oriented facets of the isolated grains. The related azimuthally averaged 1D SAXS profile shown in Fig. 5 ▸(*c*) presents many oscillations at lower *q* values and a broad scattering peak around *q* = 2.5 nm^−1^. The oscillations come from the scattering of the grain, while the broad scattering peak around *q* = 2.5 nm^−1^ contains information on the porous structure. Taking into account the polyhedral form of the grain observed by scanning electron microscopy [Fig. 5 ▸(*a*)], we simulated the scattering oscillations from the grain surface using a rectangular cuboid form factor with the density of ThO_2_ and a size of around 3 µm. The results are presented in Fig. 5 ▸(*d*). Similar spike-like patterns have already been observed with oriented crystals (Brumberger, 2013 ▸; Narayanan, 2014 ▸; Beuvier *et al.*, 2015 ▸). However, to our best knowledge, this is the first time that a combination of scattering signals from the grain surface and the internal porous structure of a highly absorbing material has been observed. Such information provides a promising route to study the alteration of the external surface and the internal surface (porosity) of highly absorbing materials at the same time, as shown by Sicard *et al.* (2004 ▸). Furthermore, we have to be cautious considering the refraction effect with dense powders. With refraction phenomena, the direct beam is broadened, as shown in the supporting information. This effect is negligible in small sample-to-detector distances (*D* = 1.2 m) and a single grain. However, this is not the case for ultra-SAXS experiments (*D* = 31 m), as shown in the supporting information.

## 4. New experimental method to obtain the absolute intensity for high-absorbing material with a laboratory SAXS instrument

Since it is not easy to have access to synchrotron facilities to perform these types of SAXS measurements, we propose a new and robust experimental method to correctly determine the scattering intensity of highly absorbing granular samples on an absolute scale using a laboratory SAXS instrument. As discussed in the introduction, with granular highly absorbing materials, the transmission can be due to a fraction of the incoming photons that do not encounter any powder in the sample cell, leading to the collection of the leaked beam, 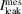 . This does not allow us to calculate the absolute scattered intensity 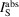 . In this part, a new simple experimental method allowing one to correct the absorption impact is proposed to determine 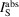 of highly absorbing porous materials from the measured scattered intensity 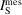 using an Mo X-ray source (*E* = 17.4 keV).

**Figure 6:**
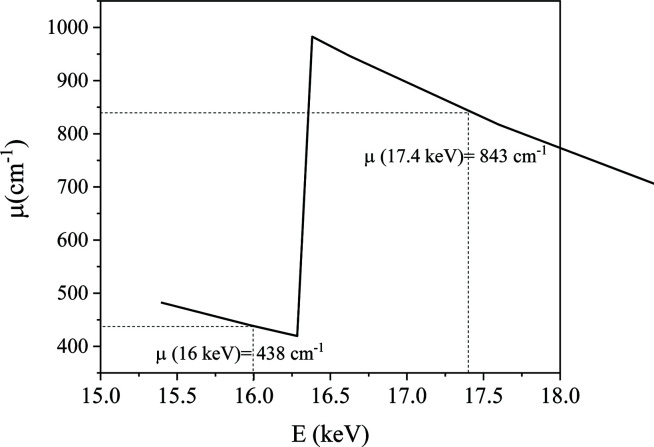
Linear attenuation coefficient μ (cm^−1^) of ThO_2_ as a function of X-ray energy *E* (keV).

By several transmission and SAXS measurements at various positions from the top to the bottom of the capillary, as described in Fig. 5 ▸(*a*), we determined the real transmission through the sample 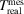 , *e.g*. when 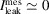 . Afterwards, 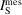 and then 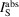 can be calculated. The acquisition of 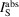 in absolute intensity allows the application of the invariant and Porod’s law for the calculation of the pore volume and the specific surface of the porous material. Then these data were compared with the measurements obtained on the ID02 beamline at ESRF (*E* = 16 keV) with a lower linear attenuation coefficient (438 versus 843 cm^−1^, as shown in Fig. 6 ▸) and a smaller beam size (70 × 90 versus 800 × 800 µm). In that case, the problems of high absorption and the leaked beam are avoided.

For measurements performed both in the laboratory and at ESRF, we used a capillary filled with a micrometric powder of ThO_2_ presenting a tailored microporosity.

**Figure 7:**
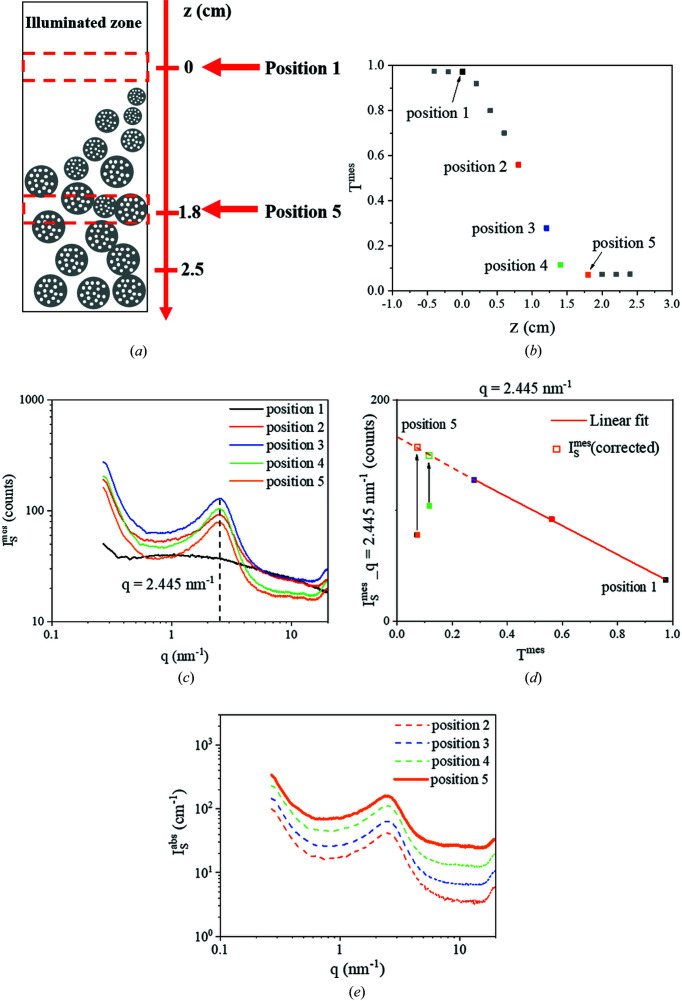
(*a*) A schematic illustration of the location of the transmission and SAXS measurements along the capillary filled with ThO_2_ powder. (*b*) Measured transmission *T* ^mes^ as a function of the vertical distance *z* from the powder/air interface. (*c*) SAXS patterns of the sample at positions 1 to 5. (*d*) Scattered intensities of the sample 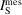 at *q* = 2.445 nm^−1^ as a function of *T* ^mes^. (*e*) SAXS patterns in absolute intensity 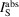 of ThO_2_ powder at different positions.

Fig. 7 ▸(*b*) presents the measured transmissions *T* ^mes^ of ThO_2_ powder [*I* ^mes^(*q*)] as a function of vertical position *z* along the capillary, as described in Fig. 7 ▸(*a*).

In order to normalize *I* ^mes^ to obtain 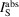 , two steps need to be carried out. First, the transmission of the sample 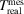 has to be determined from *T* ^mes^ without the contribution of 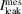 . For *z* ≥ 1.8 cm (position 5), *T* ^mes^ is constant, indicating that 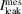 is negligible. Consequently, 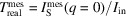 , and at position 5, 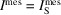 . Second, 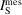 has to be corrected for the multiple-scattering and indirect absorption effects occurring in low-transmission cases. To reach this goal, 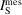 at *q* = 2.45 nm^−1^ obtained from the SAXS patterns at positions 1 to 5 [Fig. 7 ▸(*c*)] was plotted as a function of *T* ^mes^ [Fig. 7 ▸(*d*)]. The measured intensities from positions 1 to 3 can be fitted using a linear regression, which is not the case for the values at positions 4 and 5 which are less intense than the linear fit due to the multiple-scattering and indirect absorption effects. These effects were corrected using the linear-regression fit. Based on this correction, the normalization from measured intensity 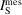 to absolute intensity 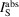 can be performed correctly using

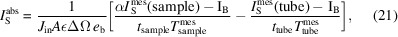

where α is a factor to correct the multiple-scattering and indirect absorption effects; *t*_sample_ and *t*_tube_ are the measuring times (s) of the sample and the empty tube, respectively; *I*_B_ is the dark count intensity; and 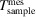 and 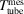 are the transmissions of the sample and the empty capillary, respectively.

Fig. 7 ▸(*e*) illustrates the corrected SAXS patterns at the five positions in absolute intensity and highlights the possible errors on 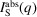 due to the contribution of 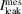 . Table 1 ▸ presents the specific surfaces Σ and the porosity values ϕ calculated from the SAXS patterns measured in the laboratory and at ESRF by Porod’s law and the invariants as shown by Spalla *et al.* (2003 ▸) and Chavez Panduro *et al.* (2012 ▸) for granular samples.

**Figure 8:**
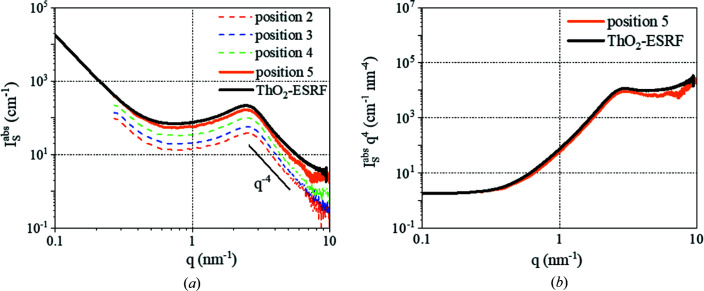
(*a*) SAXS patterns of all positions and the measurement at ESRF after background subtraction. (*b*) 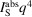 versus *q* for position 5 and the measurement at ESRF.

To calculate the specific surface, the first step is to subtract the background scattering of the patterns to reveal the Porod region [Fig. 8 ▸(*a*)]. Then Porod’s limit is obtained: for example, for position 5, 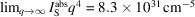 . The surface per volume can be deduced by Porod’s law:

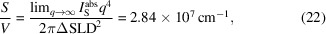

where *S* is the surface area of the sample and *V* is the volume of the sample.

This value can be finally converted to specific surface Σ by dividing by the density of ThO_2_: ρ_ThO2_ = 10 g m^−3^. In this case, Σ(position 5) = 284 m^2^ g^−1^.

The porosity was calculated with the invariant according to the method developed by Spalla *et al.* (2003 ▸) and completed by Chavez Panduro *et al.* (2012 ▸):

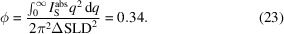

The results presented in Table 1 ▸ highlight the errors that may be induced by the contribution of 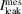 to the SAXS patterns. In this case, position 5 corresponds to the right values of Σ and ϕ of the materials. The values obtained at ESRF are comparable to those obtained in the laboratory. The slight difference can be explained by the fact that the samples were prepared in different batches.

To compare the porosity of 0.34 obtained from the invariant, we applied the disordered open connected (DOC) model to our materials, presenting a bicontinuous microporous network [Fig. 5 ▸(*a*)], to calculate the porosity. This DOC model is a structural model to analyze the scattering intensity of microemulsions with a bicontinuous cylindrical structure (Zemb, 1997 ▸) decorating a dual Voronoi network (Barnes *et al.*, 1988 ▸).

The ϕ of 0.34 calculated from the invariant is consistent with the value obtained with this model. In this case, the density of the pores *n*_pore_ can be deduced by the characteristic distance *d* = 2π/*q*_max_ = 2.43 nm:

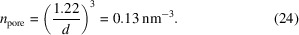

Considering the theoretical volume fraction of cylindrical pores expressed as 

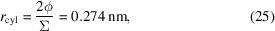

where

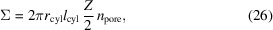


*r*_cyl_ is the average radius of the cylinders, *l*_cyl_ is the average length of the cylinders and *Z* is the average number of cylinders sharing a common vertex, then the theoretical volume fraction of the cylindrical pores is

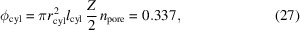

with *Z* = 2 and *l*_cyl_ = 11 nm. This value is very close to the experimental value (ϕ = 0.34) deduced by SAXS analysis. The inconsistent result obtained with spherical pores (ϕ_sph_ = 0.038) is detailed in the supporting information.

## 5. Conclusions

In this article, we have calculated the contributions of scattering and reflectivity in a signal measured by SAXS of a single grain of highly absorbing ThO_2_. According to these theoretical evaluations, the reflectivity can be neglected when the grain size is smaller than or equal to 10 µm. On the basis of these results, we have probed one isolated micrometric grain of microporous ThO_2_ and obtained the scattering signals of the grain and the bulk porous structure with negligible reflectivity signal, using a synchrotron X-ray source to cover a broader *q* range and minimize the absorption effect with a λ below the *L*_3_ edge of Th. This result provides a promising route to analyze the alteration of porous materials, determining the evolution of the internal and external surfaces. Furthermore, we proposed a new experimental method allowing the determination of scattering intensity on an absolute scale with a laboratory X-ray instrument. For the first time, to the best of our knowledge, access to the total porous texture of highly absorbing powders can be obtained without a synchrotron X-ray source. These findings open new perspectives for the characterization of nanoporous highly absorbing powder. This will expand the investigation fields to processes occurring with these particular materials, such as surface modification of powder used for catalysis application, the alteration of spent nuclear fuel in aqueous solution in the context of reprocessing and direct geological repository, and the corrosion of alloy powders used as dopants for medical implants or materials dedicated to aeronautics.

## Supplementary Material

Supporting information. DOI: 10.1107/S1600576722006987/ge5112sup1.pdf

## Figures and Tables

**Table 1:** Specific surfaces Σ and porosity values ϕ calculated from positions 2 to 5

Batch	Position	Σ (m^2^ g^−1^)	Porosity ϕ
A	2	62 ± 6	0.08
	3	79 ± 5	0.11
	4	122 ± 9	0.19
	5	242 ± 24	0.34
B	ESRF	308 ± 31	0.45
