# Supplementary material for: Specific analysis of highly absorbing nanoporous powder by small-angle X-ray scattering
Source: J Appl Crystallogr. 2022 Sep 6;55(Pt 5):1154–63. doi: 10.1107/S1600576722006987 (PMC9533747; doi:10.1107/S1600576722006987)
Supplement: Supplementary file 1 [file j-55-01154-sup1.pdf]

JOURNAL OF
APPLIED
CRYSTALLOGRAPHY

Volume 55 (2022)

Supporting information for article:

Specific analysis of highly absorbing nanoporous powder by small-angle X-ray scattering

Zijie Lu, Diane Rébiscoul, Theyencheri Narayanan and Thomas Zemb

S1. Volume fraction in the case of spherical porosity

If the pores are spherical, the average radius of the pores can be calculated as:

$$r_S = \frac{3\phi}{\Sigma} = 0.412 \text{ nm.} \qquad \text{(eq.1)}$$

In this case, the theoretical volume fraction of the spherical pores is:

$$\phi_S = \frac{4}{3}\pi r_S^3 n = 0.038. \qquad \text{(eq.2)}$$

This value is much smaller that the volume fraction (0.34) calculated by SAXS analysis. This is due to the fusion of the pores in which the pores are no longer spherical.

S2. Broadening of the direct beam due to the refraction effect

Figure S1 presents the direct beam images of the capillary and the dense powder sample with different sample-to-detector distance (D = 31 m and D = 1.2 m). When D = 31 m, the size of the direct beam through the empty capillary is 172×62 μm^2 and the one of the sample is 800×800 μm^2. This broadening effect is due to the X-ray refraction by the ThO_2 powder. The effect is not significant for an isolated grain and a large number of grains are required to produce the observed feature. In this case, one should be cautious about the contribution of refraction in the USAXS measurements. Such a broadening effect would lead to an incorrect transmission since the broadened beam size could be larger than the detecting zone of the transmission monitor. This effect should be taken into account during the merging of scattering curves with different sample-to-detector distances (Figure S2). When D = 1.2 m, the size of the direct beam of the capillary is 93×76 μm^2 and the one of the sample is 115×108 μm^2. In this case, the broadening of the direct beam is not so significant that the refraction effect can be neglected. This distance D corresponds to the range of q from 0.1 nm^{-1} to 10 nm^{-1}.

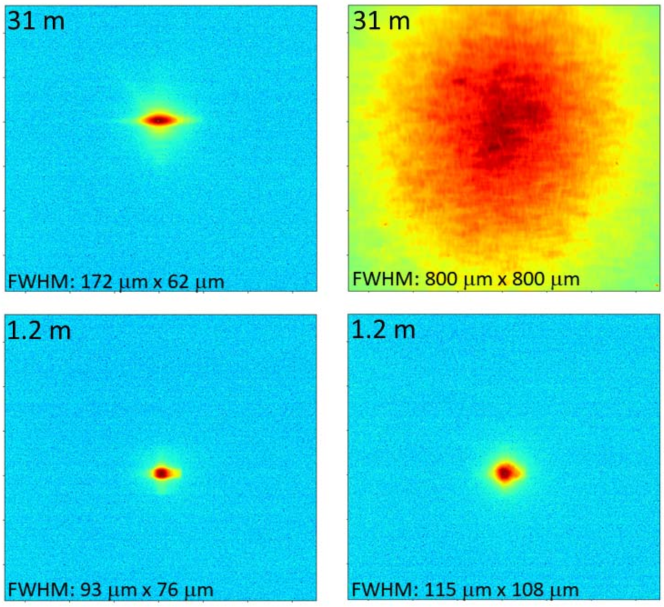

Figure S1 Direct beam images of the empty capillary and the dense powder sample with the sample-to-detector distances D = 31 m and D = 1.2 m.

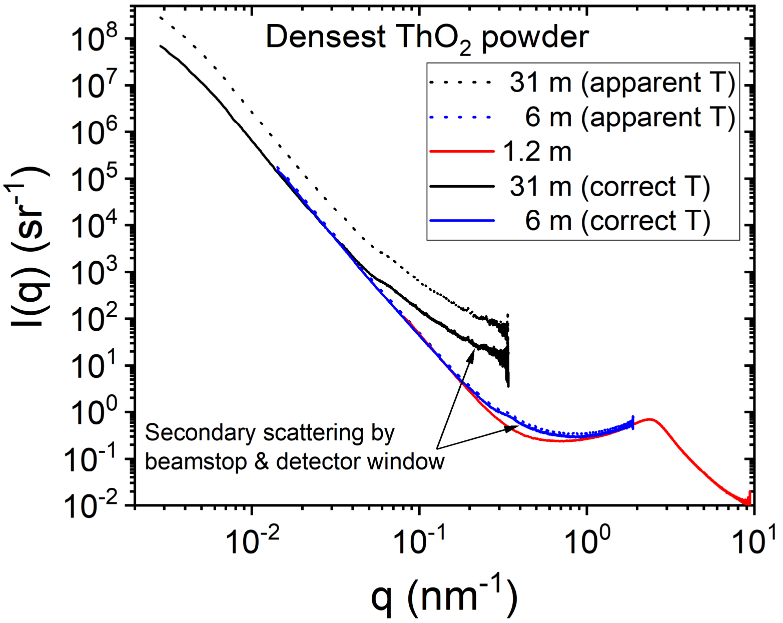

Figure S2 Merging of the scattering profiles from a dense powder sample with different sample-to-detector distances (D = 1.2, 6 and 31 m). The solid lines represent the data with the correct transmission and the dash lines represent the data with the apparent transmission at different D. The rising parts around q = 10^{-1} nm of the curves are due to the secondary scattering of the beamstop and the detector window when the beam became very broad.
